# Myocardial triglyceride content in patients with left ventricular hypertrophy: comparison between hypertensive heart and hypertrophic cardiomyopathy

**DOI:** 10.1186/1532-429X-17-S1-P246

**Published:** 2015-02-03

**Authors:** Eiryu Sai, Kazunori Shimada, Takayuki Yokoyama, Shuji Sato, Makoto Hiki, Tetsuro Miyazaki, Shigeki Aoki, Hiroyuki Daida

**Affiliations:** Cardiovascular Medicine, Graduate School of Medicine, Juntendo University, Tokyo, Japan; Radiology, Juntendo university hospital, Tokyo, Japan; Radiology, graduate school of medicine, Juntendo university, Tokyo, Japan

## Background

Proton magnetic resonance spectroscopy (^1^H-MRS) enables to assess the myocardial triglyceride (MTG) content, which is reported to be associated with cardiac dysfunction and morphology accompanied by metabolic disorder and cardiohemodynamic status. However, clinical usefulness of measurement of MTG content in patients with left ventricular hypertrophy (LVH) has not been investigated.

## Methods

To quantify MTG content, we performed ^1^H-MRS in 39 subjects with LVH. Left ventricular (LV) function was measured by cardiac magnetic resonance imaging. We divided into HHD and HCM groups on the basis of histology and/or the late gadolinium enhancement pattern.

## Results

The MTG content was significantly higher in the HHD group than in the HCM group (2.14±1.29% vs 1.09±0.70%; P=0.0009). In the HCM group, MTG content was significantly associated with LV mass (*r*=-0.41, P<0.04). In the HHD group, MTG content was significantly associated with LV end-diastolic volume, stroke volume, and cardiac output (*r*=-0.63, *r*=-0.76, *r*=-0.62, all: P<0.05).

## Conclusions

The MTG content of HCM was negatively correlated with LV mass but not LV function. These results suggested that the measurement of MTG content by ^1^H-MRS might be useful for grading HCM pattern as well as distinguishing between HCM and HHD.

## Funding

None.

